# Trends in the use of seat belts and mobile phones and their seasonal variations in Florence (2005-2015)

**DOI:** 10.1371/journal.pone.0208489

**Published:** 2018-12-11

**Authors:** Chiara Lorini, Jacopo Bianchi, Gino Sartor, Maria Grazia Santini, Anna Mersi, Saverio Caini, Guglielmo Bonaccorsi

**Affiliations:** 1 Department of Health Science, University of Florence, Florence, Italy; 2 School of Specialization in Hygiene and Preventive Medicine, University of Florence, Florence, Italy; 3 Department of Prevention, Central Tuscany Local Health Authority, Florence, Italy; 4 Department of Experimental and Clinical Medicine, University of Florence, Florence, Italy; Monash University, AUSTRALIA

## Abstract

**Introduction:**

About 1.25 million people worldwide die every year because of road accidents. Risk is higher when drivers use mobile phones, whereas seat belts help to prevent crash-related injury. We aimed to evaluate the prevalence, associated factors, and temporal trend of the use of seat belts and mobile phones among drivers and passengers in Florence, Italy (2005–2015).

**Methods:**

Use of seat belts and mobile phones use was monitored via direct observation in four areas in the province of Florence. We fitted Poisson regression models with robust variance to investigate the factors associated with the use of seat belts and mobile phones use by the drivers and to explore long-term trends and seasonal patterns in the two time-series.

**Results:**

We observed a total of an overall 134,775 vehicles: seat belts were worn by 71.8% of drivers and front-seat passengers and 27.6% of back-seat passengers, while mobile phones were being used by 4.8% of drivers. Drivers were more likely to wear seat belt when transporting passengers (≥2 vs none: prevalence ratio [PR] 1.21, 95% confidence intervals [CI] 1.14–1.29) and while driving in the afternoon (PR 1.04, 95% CI 1.03–1.05), and less likely when the front-seat passenger was not wearing seat belts (PR 0.33, 95% CI 0.32–0.34). After an initial increase, seat belts use by the driver decreased over time (-0.5% each year during 2010–2015), with significant peaks and troughs in July and January, respectively. Mobile phone use by the driver was inversely associated with wearing seat belts (PR 0.67, 95% CI 0.64–0.70) and carrying passengers (≥2 vs. none PR 0.20, 95% CI 0.07–0.52). The proportion of drivers using mobile phones did not vary over time nor showed any clear seasonality.

**Conclusions:**

Drivers’ risky behaviours (not wearing a seat belt and using a mobile phone) are associated, showing a global misperception of risk among a subset of drivers. The number of passengers and their behaviour is also associated with the driver’s attitude. The effectiveness of primary enforcement laws has declined in Italy in recent years; therefore, other strategies should be devised and implemented.

## Introduction

One of the most relevant priorities in Public Health is road accidents. Worldwide, about 1.25 million people die each year as a result of road traffic accidents, which have become one of the leading causes of death among people aged 15–29 years [1] and account for the 2.99% of the overall Disability-adjusted life years (DALYs) [2]. According to the World Health Organization (WHO) [1], these numbers are expected to increase, and road accidents are set to become the seventh leading cause of death by 2030 if there is no intervention. The urgency of facing this issue has led the WHO to draft the “Decade of Action for Road Safety 2011–2020” [3] plan and to organize the Second Global High-Level Conference on Road Safety in Brasilia in November 2015 [4], where the progress of the plan was reviewed.

In Europe, almost 85,000 people died of road traffic injuries in 2013 [5], although there was an 8.1% fall in comparison to 2010 [5]. There is a huge difference among high-income and low-income countries [5]. Among high-income countries, Italy falls in the middle ranking with 173,892 road accidents in 2015, in which 3,419 people died and 246,050 were injured [6]. Compared to the previous year, the number of accidents and injured people has decreased by a few thousand; however, deaths have increased by 1.1% [6]. Nevertheless, over the past 15 years, the number of deaths has fallen by more than 50% [6].

The risk of road accidents is higher when the driver is using mobile phones (MP) in handheld mode, especially if the driver is texting, locating the phone, or dialling [7]. The 2011 WHO report on the problem of driver distraction due to MP use [8] estimates that 1–7% of drivers use MPs while driving. A more recent German study (November 2016) reports 6.7% of drivers using MPs, 2.2% phoning in handheld mode, and 4.5% handling a smartphone (i.e. drivers had their smartphone in their hands and were operating it, looking away from the road and at the screen) [9]. Moreover, MP use and not using a seat belt (SB) are reported to be significantly associated [10].

SB is one of the most cost-effective preventive measures to reduce injuries, disabilities, and mortality caused by road accidents [1]. The prevalence of SB use varies among countries, partly depending on national regulations. In Italy, the prevalence of the use of SB is 61.2%, according to 2015 data [11]. However, we notice a north-south gradient—from 79% in northern regions to 31.7% in southern and insular areas [11]. Due to this gradient, local studies should be useful to understand this phenomenon better and more deeply. Further variability can be seen in different sampling areas—prevalence in urban areas is above 60% while outside urban areas, it is 55.2% [11].

The adoption of a specific legislation is a key factor in lowering the health consequences of road accidents. In Italy, the use of SB has been mandatory for front-seat passengers (FSP) of cars since 1989, and for all occupants since 1992 (Highway Code, Legislative Decree No 285/1992). Moreover, the use of MP in hand-held mode is not allowed while driving (Highway Code, Legislative Decree No 285/1992). the new points-based driving license, introduced by the new Highway Code in 2003 [12], is described by many authors as effective in reducing road accidents, at least in the short term [13,14]. To the best of our knowledge, no studies have been published to confirm the effect of the introduction of the points-based driving license in the long term.

The purpose of our study is to evaluate the prevalence, associated factors and temporal trend of SB use (among drivers, FSP, and back-seat passengers—BSP) and of MP use among drivers in Florence, central Italy, from 2005 to 2015.

## Materials and methods

The former Local Health Authority (LHA) of Florence has monitored the use of car safety devices and MP usage [15][16] since 2005, through the “Ulisse” system, which covers the whole of Italy [17]. Unlike in other geographical areas, the surveillance has been continuously conducted in Florence since 2005, including additional information (i.e. day of the week, time of the day). Routine monitoring was preceded by two pilot surveys, conducted in 2003 and 2004, to test the procedure [15]. The statistical units are vehicles (cars or vans). Observations were made directly by prevention technicians (health professionals responsible for carrying out measures for protecting public health, who are involved in a variety of activities focused on prevention, consultation, investigation, and education of the community regarding health risks and maintaining a safe environment), previously trained to follow a standard protocol, in four sites—one for each area of the LHA (Central, North-western, South-eastern, North-eastern). The four areas have different geographical characteristics, as shown in Table 1. To help the observer’s task, the observation sites were placed in proximity to places where vehicles had to slow down (e.g. crossroads, traffic lights, or parking entrances). No relevant viability changes were made near the observational sites during the considered period. Due to logistic issues, fewer observations were recorded in the North-eastern area. The monitoring was conducted between 7 am to 8 pm, mostly from Monday to Saturday. Data were recorded by registering the use of SB by the driver and the passengers and the use of MP by the driver on specific paper sheets, and then entered into an electronic database. The choice of the days for observation was not random but depended on the technicians’ workload. With regard to the sampling method on site, in order to minimize any risk of systematic sampling bias, technicians were instructed to observe and register the first passing vehicle after the registration of the previous vehicle. In this study, data collected between 2005 and 2015 are considered for the analysis.

**Table 1:** Characteristics of geographic areas.

*Area*	*Urbanization level (inh*^*1*^*)*	*Distance from Florence city centre*
**Centre of Florence**	Urban (380,000)	-
**North-West**	Industrial	<5 km
**South-East**	Urban (17,000)	40 km
**North-East**	Urban (18,000)	30 km

^*1*^*inhabitants of the municipality*.

Firstly, descriptive analysis was performed, using the chi square test to compare proportions.

Secondly, two binomial multivariate Poisson regression models with robust variance were fitted to estimate the association between covariates and the use of SB (first model) or MP (second model) by the driver in terms of adjusted prevalence ratios. In the first model, we included the year, the season, the time of the day, the number of passengers, the use of SB by the FSP, the use of MP by the driver, and the site of observation as covariates. In the second model, we included the use of SB by the driver as the covariate and we dichotomized the year variable, since we did not find any trend in the descriptive analysis and there was no clear difference among the years.

Thirdly, we analysed monthly trends of SB and MP usage prevalence. Since the number of observations changed over months, we obtained the percentage of SB non-use and MP use per month and then plotted the average proportion of drivers wearing SB and using the MP while driving for each month (using the 3-month moving average instead of actual data) during the study period.

We fitted additive Fourier terms (along with a term for the existence of a linear trend) into Poisson regression models with a robust variance estimator to more accurately investigate the presence of seasonal patterns in the two time-series. Fourier terms were introduced in the model, which looked for cyclical patterns with 12-month, 6-month, and 3-month periods; smaller periods were not considered based on the visual inspection of the time-series. Since both time-series appeared more disturbed in the earlier years of our study period (*see* Results *section below*), and more stationary in the following years, we decided to only use data from 2010 to 2015 for the study of seasonality.

All statistical tests were two-sided and the significance level (alpha) was set at 0.05. Data were analysed with IBM SPSS Statistic 24 and Stata 14 statistical software.

## Results

The overall number of observations during 2005–2015 was 134,775, with a maximum (15,001) observations per year in 2005 and a minimum (9,458) in 2009. On average, we observed 347.3 vehicles per day of observation (CI 95% 324.6–370.1) and around 3 days of observation per month. Overall, data on 134,775 drivers, 29,408 FSP and 2,872 BSP were collected. In Table 2, the number of observations is reported by day of the week, time of the day, and observation site for every year included in the study.

**Table 2:** Use of SB by driver, FSP, and BSP and use of MP in handheld mode by the driver by day of the week, time of day, and observation site in Florence.

		Drivers			FSP		BSP	
		N	SB use prevalence (%)	MP use prevalence (%)	N	SB use prevalence (%)	N	SB use prevalence (%)
***Day of the week***	*Monday*	24,306	70.9	4.7	5,100	69.2	449	30.3
	*Tuesday*	22,442	71.5	4.6	5,120	71.3	643	21.9
	*Wednesday*	20,202	71.2	5.3	4,825	69.9	577	30.3
	*Thursday*	23,882	74.1	4.9	5,286	72.4	405	26.4
	*Friday*	27,966	70.0	5.0	5,082	71.5	453	32.7
	*Weekend*	15,977	74.0	4.1	3,995	77.6	345	24.9
Time of the day*	*7–9*	21,753	69.3	4.8	3,345	69.4	259	35.5
	*9–12*	62,649	70.3	4.8	13,753	70.0	1,344	27.5
	*12–15*	21,786	73.1	4.6	4,492	72.6	453	28.7
	*15–18*	26,414	75.6	4.9	7,127	75.0	677	25.3
	*18–20*	1,706	81.0	5.3	497	84.3	33	60.1
***Site of observation***	*Centre*	26,585	77.6	4.7	5,093	71.9	510	37.6
	*North-West*	41,671	70.6	4.6	10,311	72.59	1,441	27.3
	*South-East*	63,387	70.9	5.1	13,504	71.7	830	22.0
	*North-East*	3,132	57.1	1.5	500	56.8	91	26.4
**Total**		**134,775**	**71.8**	**4.8**	**29,408**	**71.8**	**2,872**	**27.6**

* 467 pieces of data are missing for this variable.

**Acronyms used in the table:** *SB (Seat Belts)*, *FSP (front seat passenger)*, *MP (mobile phone)*, *BSP (back seat passenger)*.

As shown in Table 2, on average, 71.8% of both drivers and FSP used SB while only 27.6% of BSP used it. These data varied across areas (p<0.001): the highest prevalence of drivers’ SB use was in the city centre (77.6%) while the lowest was in North-east—the most peripheral area (57.1%). The use of SB was higher (p<0.001) in the afternoons and in the evenings, following a growing trend during the day. The use of SB was quite constant on different days of the week, although there was a slight increase during the weekend (p<0.001), especially among FSP. The use of MP by the drivers was lower in the weekend. No evident difference appeared in the use of MP by the drivers at different periods of the day. SB use by drivers and FSP were associated, as were SB use and MP use while driving: the prevalence of usage of SB by the driver was significantly higher when the FSP was also using it (96.6% vs 31.6%; p<0.001) and when all BSP were using it (96.4% vs 3.6%; p<0.001) and was lower when MP was being used (62.3% vs 72.3%; p<0.001).

Multivariate association between covariates and SB use while driving and multivariate association between covariates and MP use while driving are shown in Table 3. One of the main factors associated with the reduction of the use of SB by the driver is its non-use by the FSP (Prevalence Ratio (PR) 0.33; CI 95% 0.32–0.34, p<0.001). The lowest use prevalence of SB was recorded in the North-East area (PR 0.74; CI 95% 0.72–0.76, p<0.001) compared to the central area of Florence. A significantly higher prevalence of SB usage by the drivers could be observed in the afternoon (PR 1.04; CI 95% 1.03–1.05) while autumn was the season when SB was most used. The higher was the number of passengers, the higher was the prevalence of SB use by the drivers (p value for trend < 0.001).

**Table 3:** On the left side, multivariate association between covariates and SB use while driving, 2005–2015. On the right side, multivariate association between covariates and MP use while driving, 2005–2015 (Poisson regression with robust variance).

n = 134729	SB use			MP use		
		PR (CI 95%)	p		PR (CI 95%)	p
**Year**	*2005*	1		2005–2009	1	
	*2006*	1.01 [1.00–1.02]	0.113			
	*2007*	1.01 [0.99–1.02]	0.335			
	*2008*	1.03 [1.02–1.05]	< 0.001			
	*2009*	0.98 [0.97–0.99]	0.007			
	*2010*	0.94 [0.92–0.95]	< 0.001			
	*2011*	0.95 [0.93–0.96]	< 0.001	2010–2015	0.99 [0.93–1.04]	0.638
	*2012*	0.94 [0.93–0.95]	< 0.001			
	*2013*	0.93 [0.92–0.95]	< 0.001			
	*2014*	0.92 [0.90–0.93]	< 0.001			
	*2015*	0.94 [0.93–0.96]	< 0.001			
**Time of the Day**	*Morning*	1		*Morning*	1	
	*Afternoon*	1.04 [1.03–1.05]	< 0.001	*Afternoon*	1.03 [0.98–1.08]	0.259
**Use of SB by the FSP**	*Yes*	1		*Yes*	-	-
	*No*	0.33 [0.32–0.34]	< 0.001	*No*	-	-
	*No FSP*	0.80 [0.79–0.81]	< 0.001	*No FSP*	-	-
**MP Use by the Driver**	*No*	1		*No*	-	-
	*Yes*	0.88 [0.87–0.90]	< 0.001	*Yes*	-	-
**SB Use by the Driver**	*Yes*	-	-	*Yes*	1	
	*No*	-	-	*No*	0.67 [0.64–0.70]	< 0.001
**Observation Site**	*Centre*	1		*Centre*	1	
	*NW*	0.91 [0.90–0.91]	< 0.001	*NW*	1.00 [0.93–1–07]	0.924
	*SE*	0.92 [0.91–0.92]	< 0.001	*SE*	1.06 [1.00–1.14]	0.047
	*NE*	0.74 [0.72–0.76]	< 0.001	*NE*	0.28 [0.21–0.38]	< 0.001
**Number of Passengers**	*Driver only*	1	< 0.001*	*Driver only*	1	<0.001*
	*1*	1.17 [1.10–1.23]		*1*	0.37 {0.34–0.41}	
	*2*	1.19 [1.12–1.26]		*2*	0.39 [0.29–0.54]	
	*>2*	1.21 [1.14–1.29]		*>2*	0.20 [0.07–0.52]	
**Season**	*Autumn*	1		*Autumn*	1	
	*Winter*	0.96 [0.95–0.97]	< 0.001	*Winter*	1.03 [0.97–1.10]	0.308
	*Spring*	0.96 [0.95–0.97]	< 0.001	*Spring*	0.88 [0.82–0.95]	< 0.001
	*Summer*	0.99 [0.98–1.00]	0.014	*Summer*	0.87 [0.82–0.94]	< 0.001

**Acronyms used in the table:** *SB (Seat Belts)*, *MP (Mobile Phone)*, *PR (Prevalence Ratio)*, *FSP (Front Seat Passenger)*, *NW (North-west)*, *NE (North-east)*, *SW (South-west)*

*p value for trend across categories.

MP use while driving did not change significantly over the years (PR 0.99; CI 95% 0.93–1.04, p 0.638), but showed a lower use in spring (PR 0.88; CI 95% 0.82–0.95, p<0.001) and summer (PR 0.87; CI 95% 0.82–0.94, p<0.001) when compared to autumn. The highest use was in the south-eastern area, with a PR of 1.06 (CI 95% 1.00–1.14, p 0.047) compared to the centre of Florence. On the other hand, the best habit was found in north-eastern area, with a PR of 0.28 (CI 95% 0.21–0.52, p<0.001) compared to the Centre. Moreover, MP use was more frequent when the driver was alone in the car (p value for trend <0.001).

**Fig 1:**
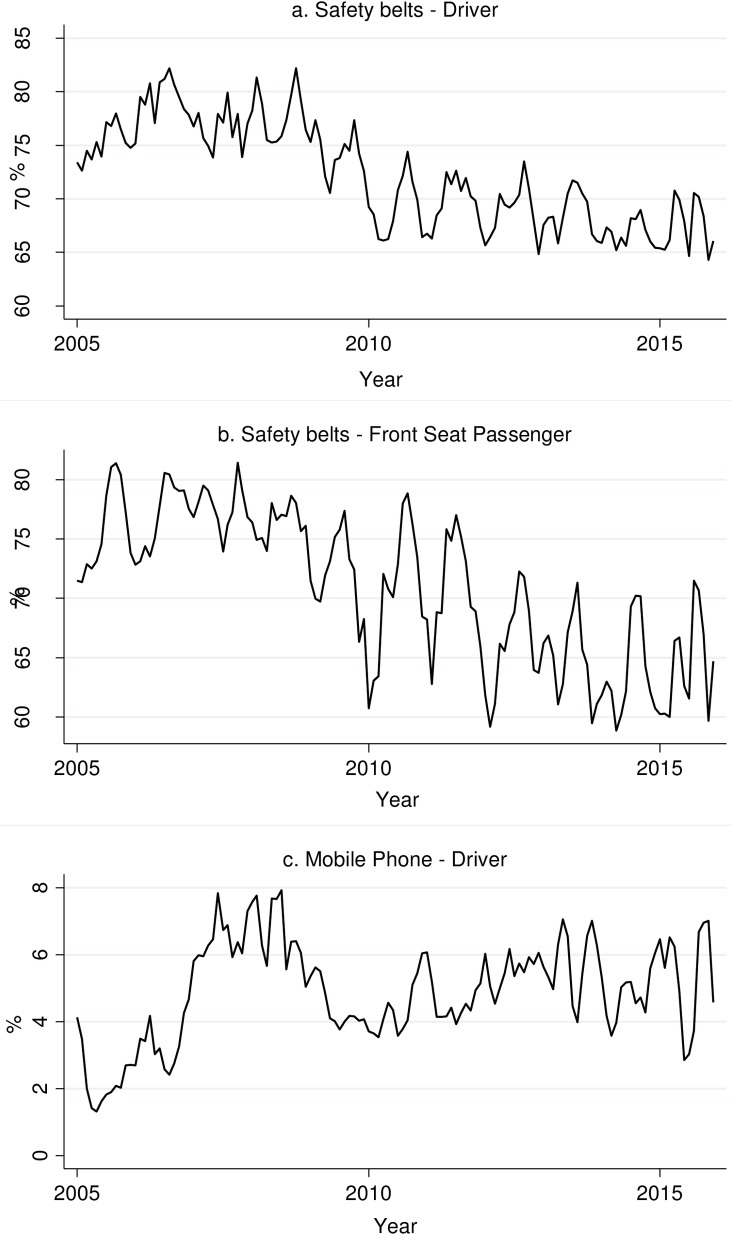
SB use by the driver (*1a*) and the FSP (*1b*) and MP (*1c*) use by the driver from 2005 to 2015 (3-month moving average).

The visual inspection of Fig 1A reveals that the use of SB among drivers increased from 2005 and stabilized around 75% during 2006–2008. It then decreased again in subsequent years, more rapidly between 2009 and 2010 (when the yearly average reached 69.3%) and followed a gradual downward slope between 2011 and 2015. The graph suggests a seasonal trend in the use of SB, with an average difference between the highest and lowest monthly values per year equal to 6.5%. An analogous trend can be seen in Fig 1B for SB use prevalence by FSP. The proportion of car drivers using a MP (Fig 1C) was at minimum values in 2005 (2.6%) and 2006 (3.6%). It subsequently increased to exceed 6% in the following two years (2007–2008), and then fell back to 4–6% between 2009 and 2015. Also, for the use of MP, peaks and troughs were evident in most years during the study period. The prevalence of the use of SB by BSP fluctuated across the years (range 16.1%– 38.9%), due to the low number of observations, and thus we excluded them from further analysis.

**Fig 2:**
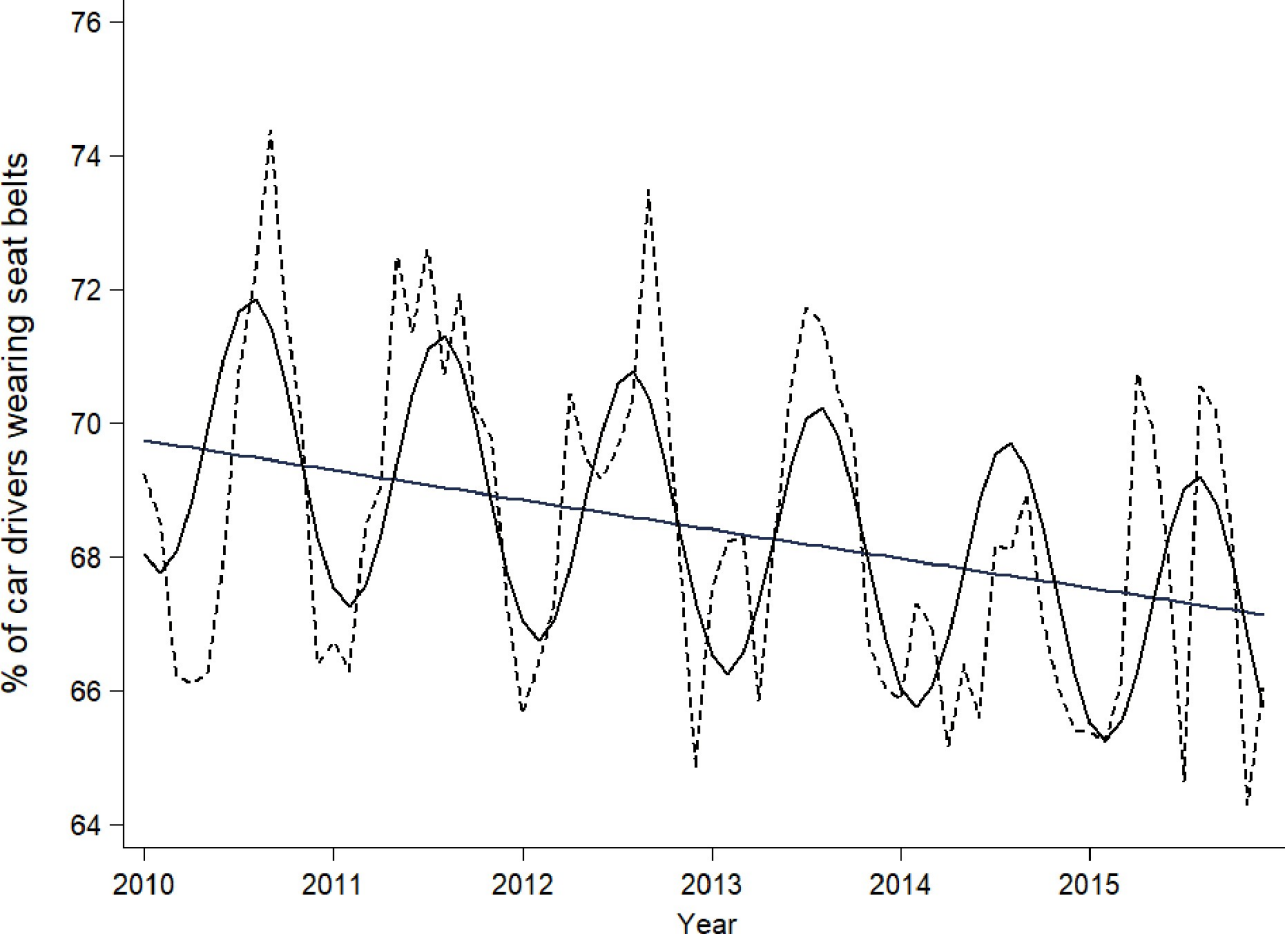
Actual (solid line) and fitted (broken line) proportion of car drivers wearing SB in the area of Florence, Italy, in 2010–2015.

Considering the period between 2010 and 2015, Poisson analysis with Fourier terms for SB use by drivers shows two main components (Fig 2). Firstly, we observed a linear trend with a mean decrease of 0.5% per year, resulting in a reduction of 3% in the whole period. Second, a periodic annual variation produced a positive peak in July and a negative peak in January, achieving a 5% amplitude. Residuals were normally distributed (skewness/kurtosis tests for normality p value = 0.85). The addition of explanatory terms to account for shorter periodicities (6- and 3-month) did not significantly improve the fit of the model.

Poisson analysis with Fourier terms for SB use by the FSP (see Supporting Information*)* also shows a linear trend and a periodic annual component.

## Discussion

Based on these results, we can make relevant considerations about the behaviour of drivers and passengers in Florence. We found a lower use of SB (71.8%) compared to other western European countries where their use is mandated by law, according to the WHO European status report on road safety [5]. On the other hand, according to “Ulisse” system data, the prevalence of SB use in our sample was higher than the Italian average (61.2%), between the average values in northern and central Italy (79.0% and 63.7% respectively in 2015) [11]. We also found differences in the four areas in our study: the prevalence of SB use was lower in the north-eastern area and higher in the centre of Florence. The lower use of SB in smaller urban areas is supported by literature [18]. This aspect should be investigated by future research both internationally and in our area. However, the use of MP was lower in the north-eastern area, probably attributable to the older population, fewer business activities and less traffic [19,20].

Our findings show that driver’s and passenger’s behaviours tend to be associated. This suggests that strategies to enhance proper behaviours should focus not only on drivers, but also on passengers. For example, educating children at primary and secondary school could have positive effects on their parents [21]. According to our results, drivers not wearing SB are more likely to use MP, showing a global misperception of risk. Prevention strategies should not address a single bad behaviour, but rather should aim to improve self-efficacy and the ability to perceive risk in daily life activities [22;23]. Driving with passengers also significantly increases SB use by the driver. Our hypothesis is that the driver may feel a greater responsibility when people are on board.

By focusing on MP use prevalence while driving, we see no trend over the years, despite the introduction of innovative devices and technologies such as Bluetooth, wireless headphones, and voice recognition software, which can reduce the need for texting and hand-held calling while driving.

We found a decreasing trend in SB use by drivers, by 0.5% per year during 2010–2015. This might be partly explained by the rebound effect of the penalty points driving in the license system, introduced in 2003 [24], which had probably increased SB use [25], albeit only temporarily. The reduction of SB use is dramatic from 2008 to 2010, which corresponds to the economic crisis period [26–28]. It is difficult to find a clear relationship between these phenomena, although literature agrees that higher socio-economic status is associated with engaging in healthy behaviours [29,30].

We found that there was high seasonal variation in SB use by drivers. In winter, drivers tended not to fasten their SB, possibly due to cumbersome clothes which make it less comfortable to wear SB. This result is somehow counterintuitive, since driving conditions in winter could be perceived as riskier because of climatic conditions (ice, fog, darkness) [31]. We also observed a higher SB compliance in the morning, as also confirmed by a study conducted in Singapore from 2011–2014 on patients attending emergency departments after road collisions [32].

Our study has some limitations. First, since the aim of the study design is to minimize misclassifications of SB and MP use, observation sites and periods were chosen using a convenience-based sampling to improve visibility and safety of observers. Sites were chosen on the basis of two pilot surveys, conducted in 2003 and 2004, to test the feasibility. The chosen roads may not be fully representative of the study area, since they had to be large and straight enough to allow the observation. Drivers could be more or less prone to using SB or MP in these streets, thus introducing a bias. Periods were instead chosen according to technicians’ workload and weather conditions, which may have introduced some bias as well. However, the number of observations in each month of the year did not substantially vary across years, and observers were instructed in order to select vehicles in a systematic way, which should minimize the risk of sampling bias affecting our findings.

The second limitation is the small number of observation points (only four in an area of 2,890 km^2^). This does not allow us to distinguish well among different road types and it could generate a sort of cluster effect since the same people tend to travel along the same road, especially in smaller municipalities. However, we consider it improbable that many vehicles were sampled more than once, given the different time periods of observation and the number of inhabitants of the municipalities.

Thirdly, the direct observation methods may have led to inaccuracies in case of limited visibility and traffic, especially for BSP and in winter (fewer daylight hours, heavier clothes). Anyway, direct observation reduced the overestimation of proper behaviour, typical of surveys using self-reported information [33]; over-reporting is frequently observed in self-reported studies on SB use, especially in low-belt-use populations, such as Italians [34, 35]. This tendency is the result of the social-desirability response bias in self-report research, which is difficult to detect or control [36]. Moreover, we could not collect information about age, sex, economic status of drivers and passengers, type of vehicle, and duration of the journey. Finally, several prevention technicians took part in the observation but interobserver reproducibility was not assessed. However, technicians were trained to standardize observations.

## Conclusions

We assessed the prevalence of SB and MP use among car drivers in Florence, Italy, during 2005–2015, and found that drivers’ risky behaviours were often combined, highlighting a general misperception of risk in a subset of drivers. In Italy, primary enforcement laws are already in force, allowing the police to stop motorists for not wearing SB. However, our data suggest that their effectiveness has declined in recent years, suggesting that alternative strategies should be devised and implemented to improve road safety. We believe it critical to adopt a public health perspective and rely on evidence-based prevention (EBP) strategies when planning interventions aiming to improve road safety. Under this regard, the Cochrane collaboration is currently assessing the effectiveness of several interventions aiming to promote the use of SB [37], such as insurance discounts, engineering-based interventions (such as alarms or restrictions in starting the car or going above a certain speed if the SB is unfastened), and education campaigns about the benefits of using SB or more generally about risk perception. We recommend that prevention strategies that have proven effective are adapted to the local context and strictly implemented in order to tackle drivers’ risky behaviours and improve road safety in Italy.

## Supporting information

S1 Fig Actual (solid line) and fitted (dashed line) proportion of front seat passengers wearing seat belts in the area of Florence, Italy, in 2010–2015. (TIF)

S1 File Lorini C, Pellegrino E, Mannocci F, Allodi G, Indiani L, Mersi A, et al. Uso delle cinture di sicurezza e del cellulare alla guida a Firenze: trend dal 2005 al 2009. Use of seat belts and mobile phone while driving in Florence: trend from 2005 to 2009. Epidemiol Prev 2012; 36 (1): 34–40.
(PDF)

S2 File Database used to perform the analysis. The variable names are a) date = date of observation; b) zona = area of observation: Central (1), North-western (2), South-eastern (3), North-eastern (4)); c) orario = time of observation: 7-10(1), 10-12(2), 12-15(3), 15-18(4), 18-20(5); 4) P1 to P5 = use of SB by the driver, front seat passenger and back-seat passengers: yes(1), no(0); 5) cell = use of mobile phone by the driver: yes(1), no(0).
(7Z) Click here for additional data file.
