# Supplementary figures and images for: Trends in the use of seat belts and mobile phones and their seasonal variations in Florence (2005-2015)
Source: PLoS One. 2018 Dec 11;13(12):e0208489. doi: 10.1371/journal.pone.0208489 (PMC6289414; doi:10.1371/journal.pone.0208489)

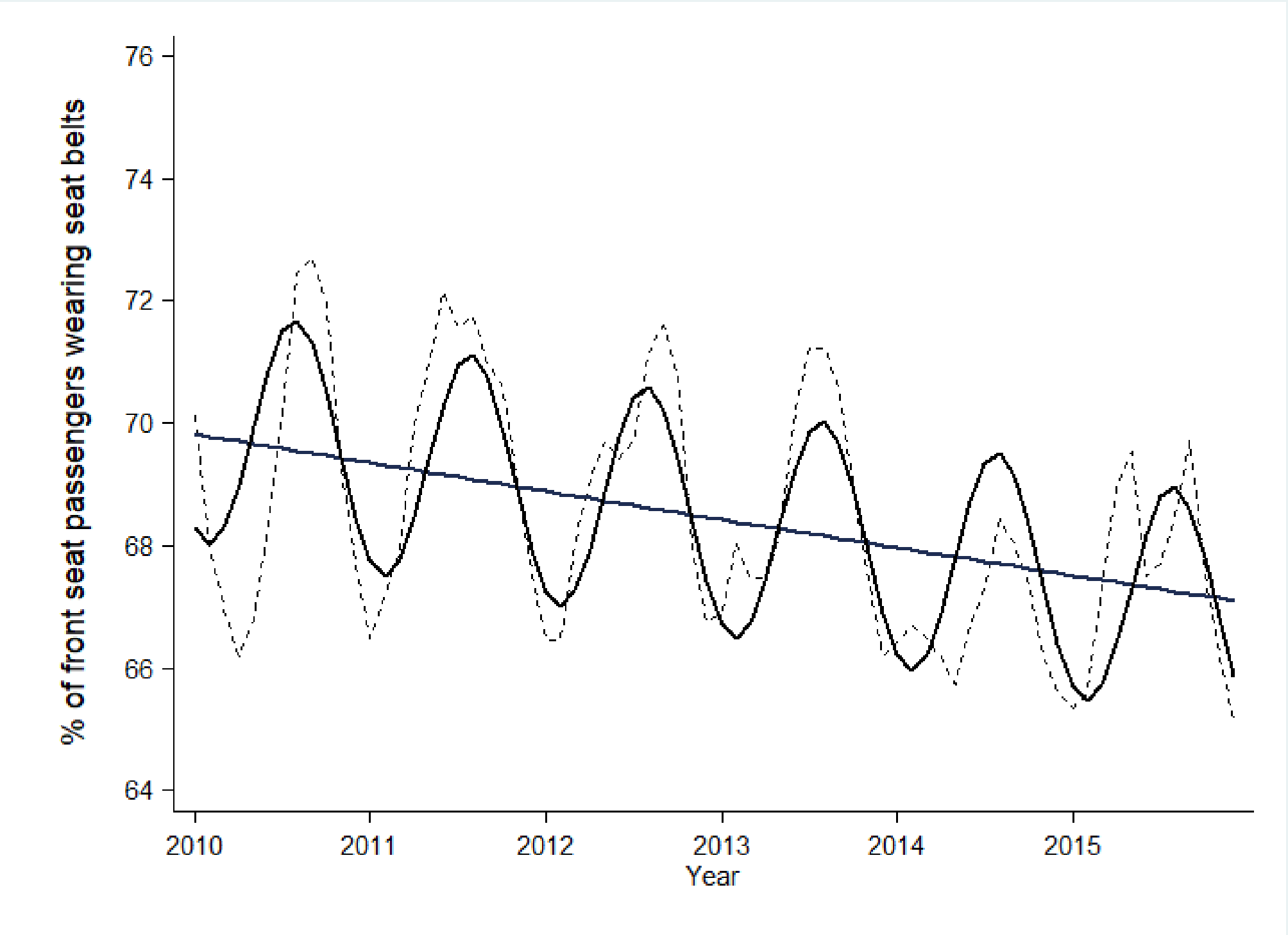

Supplement: S1 Fig — (TIF) [file pone.0208489.s001.tif]
