# Supplementary material for: Trends in the use of seat belts and mobile phones and their seasonal variations in Florence (2005-2015)
Source: PLoS One. 2018 Dec 11;13(12):e0208489. doi: 10.1371/journal.pone.0208489 (PMC6289414; doi:10.1371/journal.pone.0208489)
Supplement: S1 File — Lorini C, Pellegrino E, Mannocci F, Allodi G, Indiani L, Mersi A, et al. Uso delle cinture di sicurezza e del cellulare alla guida a Firenze: trend dal 2005 al 2009. Use of seat belts and mobile phone while driving in Florence: trend from 2005 to 2009. Epidemiol Prev 2012; 36 (1): 34–40. (PDF) [file pone.0208489.s002.pdf]

WWW.EPIPREV.IT

Uso delle cinture di sicurezza e del cellulare alla guida a Firenze: trend dal 2005 al 2009

Use of seat belts and mobile phone while driving in Florence: trend from 2005 to 2009

Chiara Lorini,[1] Elettra Pellegrino,[2] Federico Mannocci,[2] Guendalina Allodi,[2] Laura Indiani,[2] Anna Mersi,[3] Giuseppe Petrioli,[3] Maria Grazia Santini,[3] Giorgio Garofalo,[3] Guglielmo Bonaccorsi[1]

[1]Dipartimento di sanità pubblica, Università degli Studi di Firenze

[2]Scuola di specializzazione in igiene e medicina preventiva, Università degli Studi di Firenze

[3]Azienda sanitaria Firenze

Corrispondenza: Lorini Chiara, chiara.lorini@unifi.it

Epidemiol Prev 2012; 36 (1): 34-40

OBIETTIVO: valutare l'andamento temporale dell'utilizzo delle cinture di sicurezza da parte degli occupanti di autoveicoli, incluso i furgoni, e l'utilizzo del telefono cellulare alla guida senza auricolare o dispositivo di viva voce dal 2005 al 2009 a Firenze.

DISEGNO, SETTING E PARTECIPANTI E PRINCIPALI MISURE DI OUTCOME: sono state condotte osservazioni dirette (58.773 veicoli) dell'utilizzo delle cinture di sicurezza da parte degli occupanti di automobili e furgoni e l'utilizzo del telefono cellulare alla guida. E' stata valutata l'associazione tra l'uso della cintura di sicurezza da parte degli occupanti dei veicoli e tra l'uso contemporaneo di tale dispositivo e del telefono cellulare alla guida, ed è stata effettuata analisi delle serie temporali (ARIMA Box Jenkins) della prevalenza di utilizzo delle cinture di sicurezza da parte degli occupanti dei veicoli osservati, del cellulare da parte dei conducenti e il trend del rischio di guidare usando il telefono cellulare senza indossare la cintura di sicurezza rispetto a guidare usando il telefono cellulare indossando la cintura di sicurezza.

RISULTATI: le cinture di sicurezza venivano utilizzate in media dal 75,7% dei conducenti, dal 75,5% dei passeggeri anteriori e dal 25,1% dei passeggeri posteriori. La media di utilizzo del telefono cellulare alla guida è stata del 4,5%. I conducenti utilizzano più frequentemente la cintura di sicurezza se la usano anche i passeggeri anteriori e se non usano il telefono cellulare. L'utilizzo delle cinture di sicurezza da parte dei conducenti e dei passeggeri anteriori non è modificato nel tempo mentre l'uso del cellulare durante la guida è significativamente aumentato. La prevalenza di uso del cellulare alla guida tra chi non ha la cintura di sicurezza allacciata rispetto a chi la utilizza è significativamente diminuita negli anni, indicando un uso crescente di utilizzo del telefono cellulare soprattutto tra chi allaccia la cintura.

CONCLUSIONI: risulta necessario progettare e realizzare interventi di maggiore impatto su tutto il territorio in esame.

Parole chiave: cintura di sicurezza, telefono cellulare, serie temporali, correlazione.

Cosa si sapeva già

- **La prevalenza di utilizzo della cintura di sicurezza differisce tra conducenti e passeggeri di veicoli e i trend di utilizzo variano da Paese a Paese. Alcuni autori hanno inoltre dimostrato la tendenza alla contemporanea adozione di più comportamenti a rischio: l'uso delle cinture di sicurezza risulta spesso meno frequente tra coloro che usano il cellulare alla guida. Infine, in alcuni studi è descritto un costante aumento temporale dell'utilizzo del cellulare da parte dei conducenti di autovetture.**

Cosa si aggiunge di nuovo

- **Nel territorio fiorentino dal 2005 al 2009 le cinture di sicurezza sono state utilizzate in media dal 75,7% dei conducenti, dal 75,5% dei passeggeri anteriori e dal 25,1% dei passeggeri posteriori, con una tendenza alla stabilità, mentre la media di utilizzo del telefono cellulare alla guida è stata del 4,5%, con trend in crescita negli anni soprattutto tra quei conducenti che utilizzano la cintura di sicurezza. Infatti, alla fine del periodo in esame, la probabilità che un conducente senza cintura allacciata utilizzi il cellulare è praticamente la stessa di quella di un conducente con la cintura di sicurezza allacciata.**

ABSTRACT

Use of seat belt and mobile phone while driving in Florence: trend from 2005 to 2009

Epidemiol Prev 2012; 36 (1): 34-40

Objective: to evaluate the trend over time of the use of seat belts by drivers and passengers of cars and vans and the use of hand held mobile phone while driving in Florence from 2005 to 2009.
Design, setting and participants and main outcome measures: direct observations (58,773 vehicles) have been conducted to detect the use of seat belts by occupants of cars and vans, and the use of mobile phone while driving. It has been carried out correlation analysis between the use of the seat belt by occupants of vehicles and between the simultaneous use of this device and mobile phone while driving. Moreover, it has been carried out time series analysis (ARIMA Box Jenkins) of in the prevalence of the use of seat belts by occupants of vehicles observed, of mobile phone by drivers and the trend of the risk to drive using the mobile phone with unfastened seat belt rather than to drive using the mobile phone with fastened seat belt.
Results: seat belts were used on average by 75.7% of drivers, 75.5% of front passengers and 25.1% of rear passengers. The average mobile phone use while driving was 4.5%. Drivers most frequently fasten seat belt if front passengers use it and while they do not use mobile phone. The use of seat belts by drivers and front passengers has not changed over time, whereas the use of mobile phone while driving has significantly increased. The prevalence of using mobile phone with unfastened seat belt rather than to use it with fastened seat belt while driving has significantly decreased over the years, indicating an increase in the use of mobile phone, especially among those who fasten the seat belt.
Conclusions: it is necessary to plan and realize stronger interventions in the whole area.

Key words: seat belt, mobile phone, time series, correlation.

INTRODUZIONE

Gli incidenti stradali costituiscono una priorità di sanità pubblica in tutto il mondo.[1] In Europa ogni anno muoiono per questa causa circa 120 mila persone e circa 2,4 milioni rimangono infortunate. Costituiscono la prima causa di morte tra i giovani di età compresa fra i 5 e i 29 anni, con un impatto sulle economie dei singoli Paesi superiore al 3% del prodotto interno lordo.[2] In Italia, nel 2008, si sono verificati circa 220 mila incidenti stradali che hanno causato oltre 310 mila infortuni e quasi 5 mila decessi.[3] In assenza di efficaci azioni di contrasto, l'incidentalità stradale rappresenterà la quinta causa di morte nel mondo nel 2030,[1,4] la terza in termini di disabilità.[5]

Tra tutte le misure preventive volte a diminuire le conseguenze di un incidente, le cinture di sicurezza continuano a rappresentare una delle misure più efficaci, pratiche ed economiche.[1,6] La prevalenza di utilizzo di questo dispositivo varia da Paese a Paese, anche in funzione della presenza di normativa specifica. In Italia, secondo quanto rilevato con il sistema Ulisse dell'Istituto superiore di sanità, nel 2007 la percentuale d'uso della cintura di sicurezza nella parte anteriore dell'autoveicolo era del 64,6%, maggiore nel Nord (82%), rispetto al Centro (65,6%) e al Sud (46,3%).[7]

Un frequente comportamento che aumenta il rischio di incorrere in incidente stradale consiste nell'utilizzo del telefono cellulare alla guida in modalità *hand held*,[8-11] la cui prevalenza si attesta, secondo studi effettuati attraverso osservazione diretta, intorno al 3% nei Paesi anglosassoni.[12]

L'analisi condotta da Eby et al. nel 2006[13] ha rilevato, nell'arco di tempo dei quattro anni precedenti, un costante trend in aumento nell'utilizzo del cellulare da parte dei conducenti di autovetture, con un tasso medio di crescita annuo dello 0,78%. Alcuni studi hanno dimostrato, inoltre, una preoccupante tendenza alla contemporanea adozione di più comportamenti a rischio: l'uso delle cinture di sicurezza risulta infatti meno frequente tra coloro che usano il cellulare alla guida.[14]

L'introduzione di una normativa specifica è uno strumento efficace per contenere le conseguenze sanitarie degli incidenti stradali. In Italia il provvedimento legislativo più recente risale al 1° agosto 2003 con l'introduzione del nuovo codice della strada e della patente a punti, strategia che numerosi autori hanno descritto come efficace, anche in Italia, nella riduzione degli incidenti stradali e dei relativi outcome di salute, quantomeno nel breve periodo.[12,15-18]

Obiettivo del presente studio è valutare, a Firenze, l'andamento temporale dell'utilizzo delle cinture di sicurezza da parte degli occupanti di automobili e furgoni e l'utilizzo del telefono cellulare alla guida negli anni successivi all'introduzione della patente a punti.

MATERIALI E METODI

Nella Azienda sanitaria Firenze (ASF) dal 2005 vengono mensilmente condotte,[19,20] nell'ambito del sistema Ulisse,[7] rilevazioni dell'uso dei dispositivi di sicurezza. Nel presente lavoro sono stati analizzati i risultati dei dati inerenti l'uso delle cinture di sicurezza da parte del guidatore e dei passeggeri seduti sui sedili anteriori e posteriori e l'utilizzo del

cellulare alla guida in modalità *hand held* dal 2005 al 2009.
Le osservazioni vengono effettuate mediante osservazione diretta a opera di personale addetto (tecnici della prevenzione), precedentemente sottoposto a specifica formazione teorico-pratica, al fine di ottenere rilevazioni uniformi e standardizzate.
Le rilevazioni vengono eseguite in 4 distinti punti del territorio, uno per zona in cui è suddivisa l'ASF (Firenze, Nord Ovest, Sud Est, Mugello), scelti con criterio di convenienza, ovvero sulla base della possibilità di osservare i comportamenti degli occupanti dei veicoli minimizzando la possibilità di errore.
I punti di osservazione, gli stessi per tutto il periodo, sono stati i seguenti:

- Firenze: via a grande scorrimento in prossimità del centro della città;
- Nord Ovest: zona industriale ad alta intensità di traffico, in prossimità dell'ingresso di un grande centro commerciale nella prima periferia cittadina;
- Sud Est: zona urbana ad alta intensità di traffico in un comune (17.000 abitanti circa) situato a 40 km dal centro di Firenze;
- Mugello: zona urbana a media intensità di traffico in un comune rurale (18.000 abitanti circa) situato a 30 km dal centro di Firenze.

Nel periodo in studio non sono stati effettuati importanti interventi sulla viabilità in prossimità dei punti di osservazione. Nel Mugello sono emerse alcune difficoltà organizzative che hanno portato a una minor disponibilità di momenti di osservazione soprattutto in alcuni anni.
Il monitoraggio è eseguito in prossimità di luoghi sulla rete viaria dove i veicoli subiscono un rallentamento (incrocio stradale, semaforo, ingresso al parcheggio di un centro commerciale), in quattro fasce orarie con buona visibilità (mattino, primo e tardo pomeriggio), per un periodo di osservazione di un'ora durante giorni lavorativi compreso il sabato. Nel 2005 e nel 2007 è stato possibile aggiungere un'ulteriore fascia oraria (18.00-20.00) mentre alcuni problemi organizzativi hanno portato a limitare le osservazioni del sabato alle prime fasce orarie e, nel 2007, a non poter effettuare le rilevazioni in questo giorno. Per le stesse difficoltà non è stato possibile effettuare osservazioni durante la domenica. Le fasce orarie sono state individuate in modo da coprire tutte le ore di luce naturale, in modo da limitare errori di valutazione.
Gli osservatori sono presenti in coppia. Le rilevazioni interessano le vetture (autovetture e furgoni) che transitano e si soffermano nelle vicinanze degli osservatori e viene considerata una nuova vettura solamente dopo aver terminato l'osservazione della precedente.
Le informazioni, inizialmente annotate su supporto cartaceo, sono state successivamente inserite in un database elettronico.
I dati raccolti sono stati analizzati con i software statistici SPSS 18.0 e STATA 11. E' stata effettuata analisi descrittiva con analisi di *correlazione e analisi delle serie temporali.*
Le associazioni tra uso della cintura di sicurezza tra conducenti e passeggeri anteriori e tra uso, tra i conducenti, di tale dispositivo e del telefono cellulare sono state valutate mediante il test di Chi^2.
L'analisi delle serie temporali è stata condotta attraverso il modello ARIMA Box Jenkins (1,1,0) (AutoRegression Integrated Moving Average/Box Jenkins model). E' stato valutato il trend mensile della prevalenza di utilizzo del cellulare da parte dei conducenti e delle cinture di sicurezza da parte degli occupanti dei veicoli osservati. Con lo stesso metodo è stato inoltre valutato il trend mensile della prevalenza relativa di guidare usando il telefono cellulare senza indossare la cintura di sicurezza rispetto a guidare usando il telefono cellulare indossando la cintura di sicurezza, calcolato come rapporto tra la prevalenza di uso del telefono cellulare in chi non ha la cintura di sicurezza allacciata e quella di uso del telefono cellulare in chi ha la cintura di sicurezza allacciata. Per tutti i casi è stata valutata la stagionalità utilizzando il modello SARIMA (Seasonal ARIMA).
La bontà del modello ARIMA (1,1,0) è stata stimata attraverso lo studio delle funzioni di autocorrelazione (ac e pac, cioè funzione di autocorrelazione e autocorrelazione parziale) calcolate sui residui. Con il test statistico di Durbin-Watson è stato inoltre rilevato quanto l'autocorrelazione influenza il modello.
In tutte le analisi il livello alfa considerato è stato 0,05.

RISULTATI

Nel periodo 2005-2009 il numero di osservazioni nei 60 mesi indagati è risultato complessivamente di 58.773, con un massimo di 15.001 nell'anno 2005 e un minimo di 9.466 nel 2009. Il maggior numero di osservazioni è stato condotto in periferia urbana (41,2%), seguito da quelle in corrispondenza di vie a grande scorrimento (36,4%). Complessivamente sono stati osservati 15.425 passeggeri seduti sui sedili anteriori e 1.457 passeggeri collocati sui sedili posteriori.
In tabella 1 sono riportate il numero di osservazioni per giorno della settimana, fascia oraria e punto di osservazione.
Le cinture di sicurezza venivano utilizzate in media dal 75,7% dei conducenti, dal 75,5% dei passeggeri anteriori e dal 25,1% dei passeggeri posteriori (tabella 2).
La media di utilizzo del telefono cellulare alla guida è stata del 4,5%.
La prevalenza di utilizzo per mese della cintura di sicurezza da parte dei passeggeri seduti sui sedili posteriori è risultata estremamente fluttuante (range: 0-87,5%), anche a causa del numero ridotto di osservazioni, pertanto tale variabile non è stata considerata per le analisi delle serie storiche e di correlazione.
L'uso della cintura di sicurezza da parte di conducenti e passeggeri anteriori è risultato associato, così come l'utilizzo

	2005	2006	2007	2008	2009
**GIORNO DELLA SETTIMANA**					
**Lunedì**	2.148	1.596	1.124	1.111	1.796
**Martedì**	3.361	1.646	1.410	1.071	560
**Mercoledì**	1.507	2.991	1.740	2.055	2.554
**Giovedì**	3.767	2.495	2.386	2.783	1.073
**Venerdì**	2.306	2.169	4.477	3.243	2.227
**Sabato**	1.912	1.230	0	224	1.248
**FASCIA ORARIA**					
**7.00 - 9.00**	1.138	1.463	1.339	429	799
**9.00 - 12.00**	6.584	4.845	6.395	5.085	5.139
**12.00 - 15.00**	3.436	2.970	1.771	1.281	2.463
**15.00 - 18.00**	3.543	2.859	1.281	3.692	1.057
**18.00-20.00**	300	0	351	0	0
**PUNTO OSSERVAZIONE**					
**Firenze**	3.479	3.154	2.870	2.155	1.707
**Nord Ovest**	5.096	3.885	3.731	3.438	3.405
**Sud Est**	4.730	5.060	4.351	4.527	4.306
**Mugello**	1.696	38	185	367	40
Totale	**15.001**	**12.137**	**11.137**	**10.487**	**9.458**

Tabella 1. Osservazioni per giorno della settimana, fascia oraria e punto di osservazione nel territorio della ASL di Firenze nel periodo 2005-2009.
Table 1. Observations by weekday, time of day and observation point in Florence during 2005-2009.

della cintura di sicurezza e del telefono cellulare alla guida: la prevalenza di utilizzo del dispositivo di sicurezza alla guida è significativamente più elevata quando anche il passeggero anteriore lo utilizza (97,3% *vs* 36,9%; Chi^2=7199,0; p<0,001) e minore quando viene usato anche il telefono cellulare (67,2% *vs* 76,2%; Chi^2=109,0; p<0,001).
Applicando il modello ARIMA (1,1,0) SARIMA (1,1,0,12) e valutando il trend temporale si osserva che l'utilizzo delle cinture di sicurezza da parte dei conducenti dei veicoli osservati dal 2005 al 2009 non è significativamente modificato nel tempo e presenta stagionalità (p=0,034), con prevalenza generalmente maggiore in estate e autunno (figura 1).
Anche per i passeggeri seduti sul sedile anteriore, applicando il modello ARIMA (1,1,0) SARIMA (1,1,0,12) e valutando il trend temporale si osserva che l'utilizzo delle cinture di sicurezza non è significativamente modificato nel tempo. Anche in questo caso si evidenzia stagionalità (p=0.001) con prevalenza generalmente maggiore in estate e autunno (figura 1).
Applicando il modello ARIMA (1,1,0) e valutando il trend temporale si osserva che l'utilizzo del cellulare durante la guida è significativamente aumentato negli anni (coefficiente = 0,06, p<0.001) (figura 2).
Applicando il modello ARIMA (1,1,0) e valutando il trend temporale si osserva che la prevalenza relativa dell'utilizzo del cellulare alla guida tra chi non ha la cintura di sicurezza allacciata rispetto a chi la usa è significativamente diminuito negli anni (coefficiente= -0,025; p<0.001) (figura 3).
Dall'analisi grafica dei valori di ac e pac dei residui del modello ARIMA (1,1,0) applicato all'uso della cintura da parte dei conducenti, passeggeri anteriori e all'utilizzo del telefono cellulare alla guida, considerando 15 ritardi, si evidenzia che questi sono compresi nelle bande, quindi assimilabili al *white noise.*
Per ciò che attiene all'autocorrelazione, con il test statistico di Durbin-Watson è stato stimato un valore pari a circa 2 (Durbin-Watson statistic -transformed per i conducenti 2,02, per i passeggeri anteriori 2,05, per l'utilizzo del cellulare alla guida 2,27), indicando che i residui sono in media minimamente correlati e non molto differenti l'uno dall'altro. Il modello stimato dunque si adatta bene alla serie storica e il modello di regressione non dà una stima di significatività troppo elevata.

DISCUSSIONE

I risultati del presente studio consentono di formulare considerazioni preliminari sulla diffusione di alcuni comportamenti in auto nel territorio fiorentino. I limiti dei risultati sono legati alla scarsità dei punti di osservazione (solo 4 per un territorio di 2.889,6 km^2) e alla modalità di rilevazione. Infatti –sebbene l'osservazione diretta consenta di limitare la sovrastima di comportamenti corretti tipica degli studi in cui il fenomeno viene analizzato con tecniche di *self-report*[21]– in alcuni contesti (per esempio visibilità limitata, traffico sostenuto), soprattutto per i passeggeri posteriori,

Tabella 2. Utilizzo delle cinture di sicurezza da parte degli occupanti dei veicoli e del telefono cellulare alla guida nel territorio della ASL di Firenze nel periodo 2005-2009.
Table 2. Use of seat belt and mobile phone while driving in Florence during 2005-2009.

	CONDUCENTI		PASSEGGERI ANTERIORI		PASSEGGERI POSTERIORI		CELLULARE ALLA GUIDA	
	N.	PREVALENZA % DI UTILIZZO	N.	PREVALENZA % DI UTILIZZO	N.	PREVALENZA % DI UTILIZZO	N.	PREVALENZA % DI UTILIZZO
**2005**	15.001	74,2	3.694	74,8	438,0	26,6	15.001	2,6
**2006**	12.137	77,4	3.643	76,5	423,0	24,3	12.137	3,7
**2007**	11.137	76,1	2.785	77,6	251,0	35,1	11.137	6,3
**2008**	10.487	77,7	2.697	76,5	131,0	22,1	10.487	6,5
**2009**	9.458	73,4	2.606	72	214,0	14,5	9.458	4,4
**Totale**	58.220	75,7	15.425	75,5	1.457,0	25,1	58.220	4,5

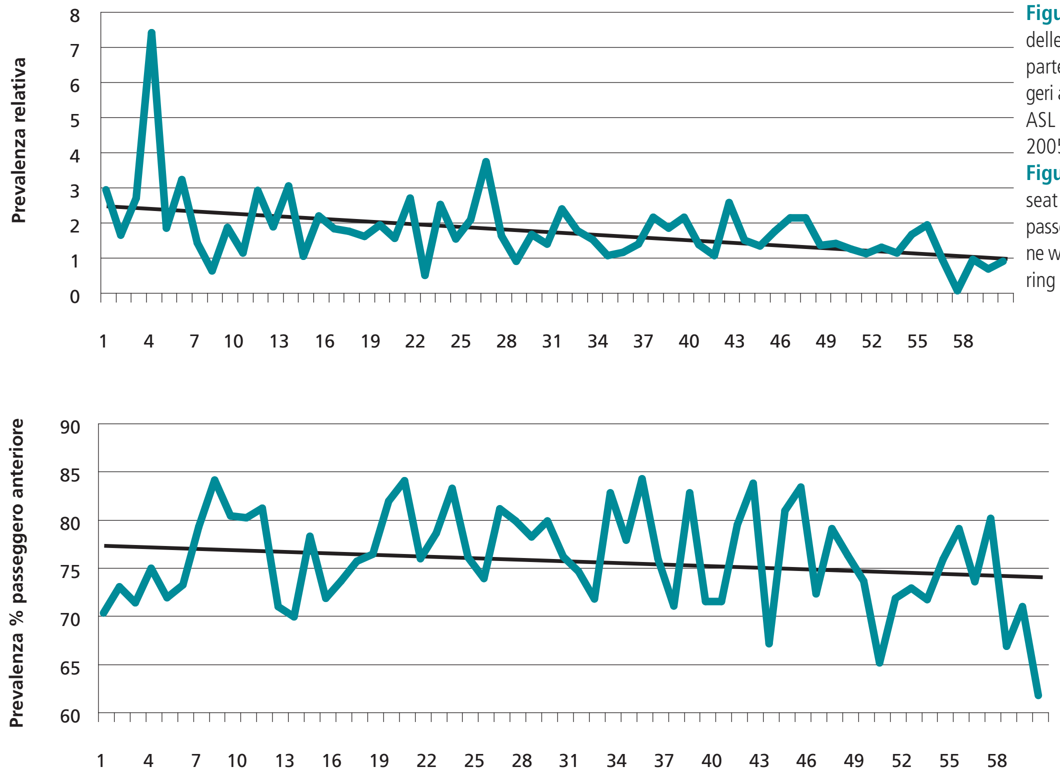

Figura 1. Trend di utilizzo delle cinture di sicurezza da parte di conducenti e passeggeri anteriori nel territorio della ASL di Firenze nel periodo 2005-2009.
Figure 1. Trend of the use of seat belt by drivers and front passengers and of mobile phone while driving in Florence during 2005-2009.

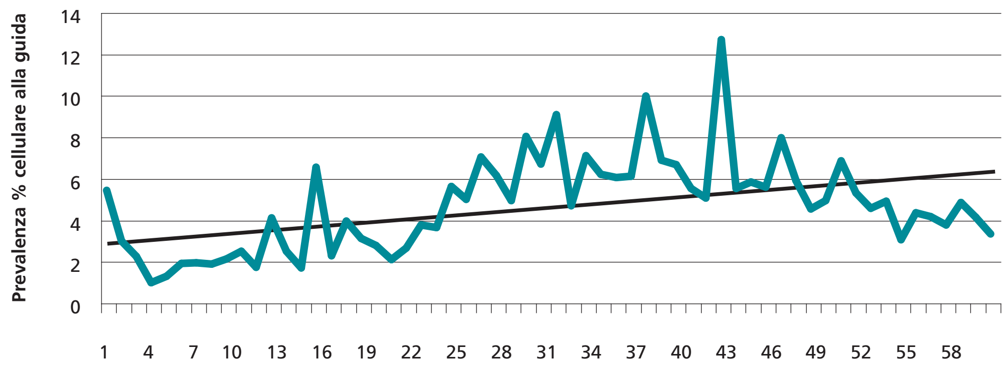

Figura 2. Trend di utilizzo del cellulare alla guida nel territorio della ASL di Firenze nel periodo 2005-2009.
Figure 2. Trend of the use of mobile phone while driving in Florence during 2005-2009.

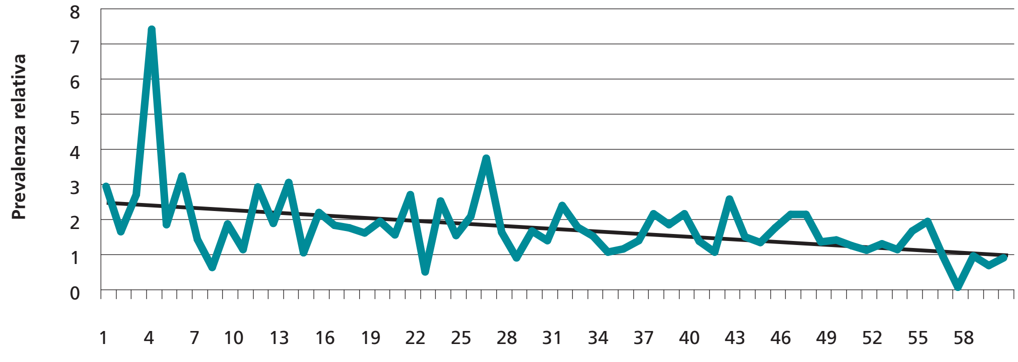

Figura 3. Trend prevalenza relativa di guidare usando il telefono cellulare senza indossare la cintura di sicurezza rispetto a guidare usando il telefono cellulare indossando la cintura di sicurezza nel territorio della ASL di Firenze nel periodo 2005-2009.
Figure 3. Trend of the relative prevalence of using mobile phone while driving with unfastened seat belt vs using mobile phone while driving with fastened seat belt in Florence during 2005-2009.

rilevare accuratamente i comportamento degli occupanti può essere difficile. Il disegno dello studio, in particolare l'individuazione dei punti di osservazione più opportuni e la definizione delle ore del giorno di osservazione, ha consentito comunque di limitare le misclassificazioni dovute a questi aspetti. Un altro fattore che influenza la qualità delle osservazioni e genera variabilità è il fatto che i rilevatori sono stati numerosi:[22] nonostante la formazione specifica finalizzata a rendere omogenee le modalità di osservazione e di rilevazione, quantomeno nei primi mesi di osservazione potrebbero essersi verificati errori di valutazione. L'aver condotto nel 2004 una prima rilevazione spot sull'uso delle cinture di sicurezza e del telefono cellulare alla guida[19] dovrebbe aver contenuto tali errori.

Nel territorio in esame la prevalenza di utilizzo della cintura di sicurezza da parte dei conducenti e dei passeggeri anteriori risulta in linea e talvolta minore a quanto riportato in letteratura per Paesi con obbligo normativo[2,22] e ad altri studi italiani,[15] minore rispetto ai Paesi europei più virtuosi, quali Austria, Svizzera, Francia, Spagna e i Paesi del Nord Europa.[2] La prevalenza di utilizzo tra i passeggeri posteriori risulta invece generalmente bassa, minore a quanto riportato in altri studi anche italiani.[2,15,16] L'uso del telefono cellulare alla guida negli ultimi anni risulta invece più diffuso rispetto a quanto riportato in letteratura per Paesi in cui non ne è consentito l'utilizzo.[12,13,14] Di fatto, dal 2005 al 2009 il 25% circa dei conducenti e dei passeggeri anteriori osservati ha viaggiato costantemente in auto senza utilizzare la cintura di sicurezza e con un uso crescente di utilizzo del telefono cellulare alla guida, nonostante il divieto normativo e la reiterata realizzazione, sul territorio fiorentino, di numerose campagne informativo-educative,[23] peraltro limitate da una diffusione "a macchia di leopardo" e un mancato coinvolgimento degli organi di polizia. Risulta pertanto necessario progettare e realizzare interventi di maggiore impatto su tutto il territorio in esame, che comprendano sì una fase di informazione, ma anche un pieno coinvolgimento degli organi deputati al controllo su strada.

Numerosi autori hanno infatti descritto come le motivazioni che più frequentemente spingono verso l'utilizzo della cintura di sicurezza siano il riconoscimento di aumento della sicurezza, l'abitudine, la certezza di andare incontro a sanzioni e i comportamenti degli altri occupanti del veicolo[24-28] mentre per quanto riguarda l'uso del cellulare alla guida la mancata percezione del rischio, l'abitudine di utilizzo e la tendenza a cercare continuamente momenti di comunicazione con gli altri.[29,30] L'associazione tra i comportamenti degli occupanti il veicolo è emersa anche nel nostro studio, ove è stata evidenziata una forte positiva tra l'uso della cintura di sicurezza da parte del conducente e del passeggero posteriore. Dai risultati emerge anche un'associazione significativa tra uso della cintura di sicurezza e telefono cellulare alla guida, sebbene i comportamenti non siano così marcatamente differenti come si osserva valutando l'utilizzo della cintura di sicurezza da parte del conducente rispetto al passeggero. Si osserva inoltre un trend di crescita d'uso del cellulare alla guida anche tra quei conducenti che utilizzano la cintura di sicurezza –alla fine del periodo in esame la probabilità che un conducente senza cintura allacciata utilizzi il cellulare è praticamente la stessa di quella di un conducente con la cintura di sicurezza allacciata– a indicare, probabilmente, una diffusa errata percezione del rischio anche tra chi abitualmente assume comportamenti corretti (esempio, utilizzo della cintura).

Un altro fattore indicato in letteratura come importante nel ridurre la prevalenza di utilizzo della cintura di sicurezza è il discomfort legato all'uso,[24] motivazione che potrebbe spiegare la tendenza, osservata nel nostro studio, a rilevare una prevalenza più bassa in inverno quando l'ingombro degli abiti è maggiore.

Interventi complessi, educativi e repressivi, rivolti soprattutto a promuovere comportamenti corretti tra i conducenti, se ben condotti e sufficientemente sviluppati nel tempo e nello spazio sono risultati efficaci, in numerosi contesti, a modificare comportamenti in strada facendo diventare routinario un comportamento inizialmente indotto da ragioni temporanee, quali la certezza di incorrere in sanzioni.[24] Il sistema di monitoraggio dei comportamenti alla guida sviluppato negli anni nel territorio fiorentino, ormai giunto a regime, potrebbe risultare estremamente utile anche per verificare l'efficacia degli interventi preventivi nello specifico contesto geografico.

Conflitti di interesse dichiarati: nessuno.

Ringraziamenti. Si ringraziano i tecnici della prevenzione che hanno effettuato le rilevazioni e il Dottor Alberto Baldasseroni per i suggerimenti per l'analisi dei dati.

BIBLIOGRAFIA

1. World Health Organization. *Global status report on road safety. Time for action.* WHO, Geneva, 2009.
2. World Health Organization Europe. *European status report on road safety. Towards safer road and healthier transport.* WHO, Copenhagen 2009.
3. Istituto Nazionale di Statistica. *Incidenti stradali*. Anno 2008. Disponibile su: http://www.istat.it/salastampa/comunicati/non_calendario/20091113_01/testointegrale20091113.pdf
4. World Health Organization. *World health statistics 2008*. WHO, Geneva, 2008.
5. World Health Organization. *The Global Burden of Diseases, 2004 update.* WHO, Geneva 2008. Disponibile su: http://www.who.int/healthinfo/global_burden_disease/2004_report_update/en/index.html
6. Kononen DW, Flannagan CAC, Wang SC. Identification and validation of a logistic regression model for predicting serious injured associated with motor vehicle crashes. *Accid Anal Prev* 2011;43:112-122.
7. Taggi F, Dosi G, Marturano P. *Il Sistema Ulisse per il monitoraggio dell'uso delle cinture di sicurezza e del casco in Italia, anno 2007*. Disponibile su: www.iss.it
8. De Jong MJ. Cellular telephone use while driving: growing awareness of the danger. *J Emerg Nurs* 2003;29:578-581.
9. Collet C, Guillot A, Petit C. Phoning while driving I: a review of epidemiological, psychological, behavioural and physiological studies. *Ergonomics* 2010;53:589-601.
10. Collet C, Guillot A, Petit C. Phoning while driving II: a review of driving conditions influence. *Ergonomics* 2010;53:602-616.
11. Ishigami Y, Klein RM. Is a hands-free phone safer than a handheld phone? *J Safety Res* 2009;40:157-164.
12. McCartt AT, Braver E, Geary LL. Drivers' use of handheld cell phones before and after New York State's cell phone law. *Prev Med* 2003;36:629–635.
13. Eby DW, Vivoda JM, St. Louis RM. Driver hand-held cellular phone use: A four-year analysis. *J Safety Res* 2006;37:261–265.
14. Eby DW, Vivoda JM, Driver hand-held mobile phone use and safety belt use. *Accid Anal Prev* 2003;35:893–895.
15. Zambon F, Fedeli U, Visentin C, Marchesan M, Avossa F, Brocco S, Spolaore P. Evidence-based policy on road safety: the effect of the demerit points system on seat belt use and health outcomes. J Epidemiol Community Health 2007;61:877-881.
16. Zambon F, Fedeli U, Milan G, Brocco S, Marchesan M, Cinquetti S, Spolaore P. Sustainability of the effects of the demerit points system on seat belt use: A region-wide before-and-after observational study in Italy. *Accid Anal Prev* 2008;40:231-237.
17. Farchi S, Chini F, Rossi PG, Camilloni L, Borgia P, Guasticchi G. Evaluation of the health effects of the new driving penalty point system in the Lazio Region, Italy, 2001–4. *Inj Prev* 2007;13:60-64.
18. Johal S, Napier F, Britt-Compton J, Marshall T. Mobile phones and driving. *J Public Health* 2005;27:112-113.
19. Lorini C, Bonaccorsi G, Mersi A, Baroncini O, Ciampi G, Boddi V, Santini MG, Comodo N. Utilizzo del telefono cellulare alla guida: l'esperienza fiorentina. *Ann Ig* 2006;18:349-356.
20. Lorini C, Bonaccorsi G, Mersi A, Petrioli G, Postiglione M, Boddi V, Santini MG, Comodo N. Utilizzo del telefono cellulare alla guida nel territorio fiorentino: i risultati della nuova indagine. *Ann Ig* 2007;19:275-280.
21. Zambon F, Fedeli U, Marchesan M, Schievano E, Ferro A, Spolaore P. Seat belt use among rear passengers: validity of self-reported versus observational measures. *BMC Public Health* 2008;8:233.
22. Majumdar A, Noland RB, Ochieng WY. A spatial and temporal analysis of safety-belt usage and safety-belt laws. *Accid Anal Prev* 2004;36:551-560.
23. Mersi A, Santini MG, Petrioli G, Lorini C, Bonaccorsi G, Comodo N. Sicurezza stradale. Attività informativo-educativa per la prevenzione degli esiti degli incidenti stradali a Firenze. *Toscana Medica* 2009; 10:15-16.
24. im eko lua Ö, Lajunenb T. Why Turks do not use seat belts? An interview study. *Acc Anal Prev* 2008;40:470-478.
25. Nambisan SS, Vasudevan V. Is seat belt usage by front seat passengers related to seat belt usage by their drivers? *J Safety Res* 2007;38:545-555.
26. Ali M, Haidar N, Ali MM, Maryam A. Determinants of seat belt use among drivers in Sabzevar, Iran: a comparison of theory of planned behavior and health belief model. *Traffic Inj Prev* 2011;12:104-109.
27. Tavafian SS, Aghamolaei T, Gregory D, Madani A. Prediction of seat belt use among Iranian automobile drivers: application of the theory of planned behavior and the health belief model. *Traffic Inj Prev* 2011;12:48-53.
28. Chliaoutakis EJ, Gnardellis C, Drakou I, Darviri C, Sboukis V. Modelling the factors related to seatbelt use by the young drivers of Athens. *Acc Anal Prev* 2000;32:815-825.
29. Walsh SP, White KM, Cox S, Young RMcD. Keeping in constant touch: the predictors of young Australians' mobile phone involvement. *Computers in Human Behavior*, 2011; 27:333-342.
30. Laws J. We can't quit. *Occup Health Saf* 2010;79:74.
